# Development and Validation of a Risk Prediction Model for Venous Thromboembolism in Lung Cancer Patients Using Machine Learning

**DOI:** 10.3389/fcvm.2022.845210

**Published:** 2022-03-07

**Authors:** Haike Lei, Mengyang Zhang, Zeyi Wu, Chun Liu, Xiaosheng Li, Wei Zhou, Bo Long, Jiayang Ma, Huiyi Zhang, Ying Wang, Guixue Wang, Mengchun Gong, Na Hong, Haixia Liu, Yongzhong Wu

**Affiliations:** ^1^Chongqing Key Laboratory of Translational Research for Cancer Metastasis and Individualized Treatment, Chongqing University Cancer Hospital, Chongqing, China; ^2^Digital Health China Technologies, Co., Ltd., Beijing, China; ^3^MOE Key Laboratory for Biorheological Science and Technology, State and Local Joint Engineering Laboratory for Vascular Implants, College of Bioengineering, Chongqing University, Chongqing, China

**Keywords:** machine learning, random forest, lung cancer, venous thromboembolism, risk prediction model

## Abstract

**Background:**

There is currently a lack of model for predicting the occurrence of venous thromboembolism (VTE) in patients with lung cancer. Machine learning (ML) techniques are being increasingly adapted for use in the medical field because of their capabilities of intelligent analysis and scalability. This study aimed to develop and validate ML models to predict the incidence of VTE among lung cancer patients.

**Methods:**

Data of lung cancer patients from a Grade 3A cancer hospital in China with and without VTE were included. Patient characteristics and clinical predictors related to VTE were collected. The primary endpoint was the diagnosis of VTE during index hospitalization. We calculated and compared the area under the receiver operating characteristic curve (AUROC) using the selected best-performed model (Random Forest model) through multiple model comparison, as well as investigated feature contributions during the training process with both permutation importance scores and the impurity-based feature importance scores in random forest model.

**Results:**

In total, 3,398 patients were included in our study, 125 of whom experienced VTE during their hospital stay. The ROC curve and precision–recall curve (PRC) for Random Forest Model showed an AUROC of 0.91 (95% *CI*: 0.893–0.926) and an AUPRC of 0.43 (95% *CI*: 0.363–0.500). For the simplified model, five most relevant features were selected: Karnofsky Performance Status (KPS), a history of VTE, recombinant human endostatin, EGFR-TKI, and platelet count. We re-trained a random forest classifier with results of the AUROC of 0.87 (95% *CI*: 0.802–0.917) and AUPRC of 0.30 (95% *CI*: 0.265–0.358), respectively.

**Conclusion:**

According to the study results, there was no conspicuous decrease in the model’s performance when use fewer features to predict, we concluded that our simplified model would be more applicable in real-life clinical settings. The developed model using ML algorithms in our study has the potential to improve the early detection and prediction of the incidence of VTE in patients with lung cancer.

## Introduction

Lung cancer is the most common incident cancer with a high mortality rate in China (1). Venous thromboembolism (VTE), which includes both deep vein thrombosis (DVT) and pulmonary embolism (PE), is the second leading cause of death in cancer patients and the overall mortality increased in cancer patients with VTE (2). Epidemiological research has found that 20–30% of first-time VTE cases are related to cancers, and the incidence of VTE in oncological patients is 4–7 times higher than that in non-tumor patients (3). The incidence of VTE in patients with cancer varies with cancer type, stage, and aggressiveness (4). In cohort studies, the incidence of VTE in patients with lung cancer receiving chemotherapy was variously reported as 16.8% at 3 months and 14.1% at 6 months after the start of chemotherapy (5). Having a VTE is a significant predictor of death within 2 years in patients with primary lung cancer, with hazard ratios (*HRs*) of 2.3 (95% *CI*: 2.2–2.4) and 1.5 (95% *CI*: 1.3–1.7) for non-small cell lung cancer (NSCLC) and small cell lung cancer (SCLC), respectively (6). Considering the severity of VTE, several medical scores for predicting risk have been designed: Khorana, Caprini, Vienna CATS, PROTECHT, and COMPASS-CAT. However, these risk prediction scores have been developed with the condition limitation at that time, and barely externally validated in patients with lung cancer. For example, the most recommended Khorana score (KS) is strongly dependent on tumor type and does not consider treatment-related factors influencing VTE development and has a low sensitivity of 10–25%, and a high specificity of 76–100% in patients with lung cancer (7).

Furthermore, as large amounts of rapidly increasing patient data can be obtained easily, it is possible to design more precise models for disease diagnosis or risk prediction. Machine learning (ML) plays an important role in analyzing complex medical data, and its superiority has been shown in studies on omics, electronic health records, and image processing (8, 9). VTE risk assessment using ML methods has been reported by several studies, but there still exist some limitations. Ferroni used data from 94 cancer patients with VTE to train the multiple kernel ML model and validated the model in a cohort consisting of 43 VTE patients and 565 non-VTE patients. However, their focus was only on cancer patients with VTE, and the sample size was small (10). Wang chose 188 patients with VTE and 188 without VTE to train multiple ML models and validated the model in a cohort consisting of 42 VTE patients and 1,537 non-VTE patients. However, the study concluded that their sensitivities are relatively low (11). Most ML models that are currently reported to predict VTE have limitations. For example, the sample size of most of these studies has been limited, and the validation of models on real clinical data is lacking. In addition, most studies (12, 13) used simply the area under the receiver operating characteristic curve or finite indicators such as precision and recall as the evaluation standard, which tends to hide some disadvantages of ML models and can hardly explain the overall model comprehensively. In addition, though some assessment tools have been designed to predict the risk of VTE for lung cancer patients, several reports demonstrated that it might not be suitable in specific local populations, such as in the case of ambulatory patients with lung cancer (14) or in patients undergoing outpatient chemotherapy for lung cancer (7).

Therefore, this study aimed to develop and validate ML models that predict the incidence of VTE among Chinese patients of lung cancer, and improve on the limitations in those studies as much as possible and design a more comprehensive and robust ML model that is suitable for lung cancer patients.

## Materials and Methods

### Patient Dataset

Data were collected from Chongqing University Cancer Hospital of China, an oncology-specific Grade 3A institution. Inclusion criteria were as follows: (1) Patients aged ≥18 years; (2) A record of at least one time of hospitalization; (3) With newly diagnosis of lung cancer by pathology; (4) Index date from January 2013 to December 2019. The exclusive criteria listed as (1) VTE had occurred before the diagnosis of lung cancer and (2) died within 48 h after admission. All available data on the database were used to maximize the power and generalizability of the results. Those patients with incomplete information were not included. Patients with multiple hospitalizations were only counted once.

We then verified outliers manually and corrected them. The primary endpoint was the diagnosis of VTE during index hospitalization. The presence or absence of VTE was decided blinded to the predictor variables since the data were collected retrospectively. Investigators, blinded to both predictor variables and outcome, reviewed and classified standardized data collection forms. Risk factors were chosen based on known risk variables and those previously described in the literature. We chose the Karnofsky Performance Status (KPS) as a performance metric. The following variables were taken into consideration: patient-related factors: age, sex, weight, height, KPS, comorbidities (chronic obstructive pulmonary disease [COPD], varicosity), VTE history, history of malignant tumor other than lung cancer; cancer-related factors: tumor stage, pathological classification, clinical stage (according to AJCC 8th edition staging system) (15); treatment-related factors: anticancer drugs (mitomycin, epithelial growth factor receptor tyrosine kinase inhibitors [EGFR-TKI], platinum-based drugs, bevacizumab, and recombinant human endostatin), central venous catheter cannulation (CVC) *via* internal jugular vein cannulation, subclavian vein, and femoral vein; and biomarker: platelet count, albumin concentration, hemoglobin, creatinine, platelet count, leukocyte count, and D-dimer. All laboratory tests were performed within 90 days before treatment. In the case of repeated tests, the results closest to the time of the treatment were used. We diagnosed VTE and comorbidities using ICD-10 codes.

### Data Preprocessing

First, missing values were treated differently for numerical and categorical features. Missing values in numerical features were imputed iteratively with the Bayesian ridge regression estimator; while for all categorical features, missing values were considered and treated as additional categories. After that, the one-hot-encoder was performed on categorical variables like “pathological type” to get a binary representation, while an ordinal encoder was used on categorical features like “stage of cancer.” Numerical variables were then scaled to zero mean and unit variance. All continuous variables were described by their count, mean, and standard deviation, while categorical variables are described by their count.

### Learning Algorithm

We divided the data into training (64%), validation (16%), and test (20%) sets randomly. Random splitting maximizes the assurance that the results of the validation and training sets are unbiased when evaluating the model. To account for the imbalance between target classes, the Synthetic Minority Over-sampling (SMOTE) algorithm was utilized at the training stage.

Then several models were compared with their performance (such as Random Forest, Adaboost, K-Nearest Neighbor [KNN], Logistic regression, and XGboost). A best-performed classifier was selected to make predictions, in our case, it was Random Forest.

Hyperparameters were tuned with Bayesian optimization on our validation set to avoid overfitting. An entire process of 5-fold cross-validation was performed with each set of hyperparameters and used the validation set to evaluate the hyperparameters. Since we aimed to correctly predict more patients with VTE, the true positive rate was valued slightly more during hyperparameter tuning by weighting it more heavily in the objective function that the Bayesian optimizer was trying to minimize. We combined the previous training and validation sets to form a new training set to train the final model with the optimal hyperparameters we found after the hyperparameter tuning.

We also investigated feature contributions during the training process with both permutation importance scores and the impurity-based feature importance scores in the selected model. The impurity-based feature importance score makes it easy to gauge each feature’s contribution. This score is based on the number of times a feature is used as a node and how much that mode reduces entropy. In conjunction with permutation importance (16), which measures the loss of model accuracy when a feature is randomly shuffled, we can obtain a reliable feature importance ranking.

For the reason that an effective clinical prediction model requires fewer features to predict, we simplified the model features based on features ranking, literature review, and clinical experiences to achieve the best balance between model performance and model real clinical applicability.

### Evaluation Methods

To evaluate the power of the ML models, we calculated and compared the area under the receiver operating characteristic (AUROC) curves. Since AUROC can be interpreted as the probability that the model would rank a randomly selected patient with VTE higher than a randomly selected patient without VTE, the higher the AUROC, the better the model’s performance (17). However, it is known that compared to the area under the precision–recall curve (AUPRC), AUROC might be misleading because it can sometimes be too optimistic (18, 19). Since our dataset is highly imbalanced, we also calculated AUPRC (also called average precision) to compensate for AUROC’s limitation. Average precision was calculated by taking the sum over the precisions achieved at each threshold multiplied by the increase in recall from the previous threshold (20). AUPRC considers the trade-off between precision and recall, thus it provides insight into how the model can accurately predict patients with VTE without falsely predicting too many patients without VTE as having VTE (19).

At the same time, test accuracy, sensitivity and specificity were also recorded. The 95% *CI* was also calculated for each performance assessment measurement to account for the uncertainty of the model by bootstrapping a sample (60%) from the training set and test set 1000 times with replacements.

## Results

### Patient Characteristics

In total, 3,398 patients were included in our analysis, 125 of whom experienced VTE during their hospital stay. The variables’ characteristics and the statistical analysis between the non-VTE and VTE subgroups are summarized in Table 1. Those who experienced VTE were, on average, younger and more likely to be male and more likely to occur in stage of IV and NSCLC patients. There were a higher proportion of patients with comorbidities, history of malignant tumor, CVC, mitomycin, recombinant human endostatin, EGFR-TKI, Platinum-based chemotherapy, and Bevacizumab in patients who had VTE compared to patients without VTE. Additionally, the detailed data distribution of categorical data, data distribution of continuous data and missing values are shown in Supplementary Material.

**TABLE 1:** Patient demographics and clinical characteristics.

Characteristic	Modifier	All *n*(%)	No VTE *n*(%)	VTE n(%)	*P*-value
Age (*n* = 3398)	years	64.03 ± 10.31	64.08 ± 10.30	62.64 ± 10.48	0.134
KPS (*n* = 3379)		76.80 ± 10.97	76.76 ± 11.05	77.88 ± 8.76	0.166
Weight (*n* = 2664)	kg	59.61 ± 10.64	59.61 ± 10.65	59.23 ± 1056	0.830
Height (*n* = 2738)	cm	161.13 ± 8.07	161.16 ± 8.06	160.32 ± 8.44	0.335
PLT count (*n* = 3349)	*10^9^/L	222.19 ± 99.73	222.52 ± 100.03	213.83 ± 91.88	0.303
Albumin (*n* = 3333)	g/L	38.84 ± 6.24	38.88 ± 6.20	37.77 ± 7.09	0.087
D-dimer (*n* = 3282)	mg/L	2.09 ± 3.65	2.05 ± 3.62	2.96 ± 4.18	0.019
Hemoglobin (*n* = 3347)	g/L	121.75 ± 20.53	121.86 ± 20.48	118.94 ± 21.67	0.141
Leukocyte count (*n* = 3350)	*10^12^/L	5.55 ± 5.35	5.51 ± 5.32	6.54 ± 6.04	0.063
Creatinine (*n* = 3338)	umol/L	66.75 ± 37.90	66.86 ± 38.34	64.04 ± 23.88	0.211
**Sex (*n* = 3357)**					
Female		1015	958	57	<0.001
Male		2342	2274	68	
**Pathological type (*n* = 2043)**					
NSCLC		1815	1724	91	0.007
SCLC		228	225	3	
**Stage of cancer (*n* = 1489)**					
I		91	90	1	<0.001
II		80	79	1	
III		329	318	11	
IV		989	887	102	
**Comorbidities**					
VTE history		172	74(2.26)	98(78.4)	<0.001
Varicosity		21	16(0.49)	5(4)	<0.001
COPD		666	633(19.34)	33(26.4)	0.065
History of malignant tumor		49	47(1.44)	2(1.6)	0.701
CVC		110	96(2.93)	14(11.2)	<0.001
**Drug utilization**					
Mitomycin		8	7(0.21)	1(0.8)	0.259
Recombinant human endostatin		108	102(3.11)	6(4.8)	0.291
EGFR-TKI		403	348(10.63)	55(44)	<0.001
Platinum-based chemotherapy		647	602(18.39)	45(36)	<0.001
Bevacizumab		78	60(1.83)	18(14.4)	<0.001

*KPS, Karnofsky performance status; PLT, platet; NSCLC, non-small cell lung cancer; SCLC, small cell lung cancer; COPD, chronic obstructive pulmonary disease; CVC, central venous catheter cannulation; EGFR-TKI, Epithelial growth factor receptor tyrosine kinase inhibitors. The P-values for all the numerical variables were calculated using Welch’s t-test. The Mann-Whitney U test was used to determine the P-value for “Stage of cancer,” and Fisher’s exact test was used to obtain the P-values for all other categorical features.*

### Model Performance

As shown in Table 2, the performance comparison of different models and their 95% CI demonstrated that the Random Forest model outperformed all other models on both AUROC and AUPRC. Although the XGBoost and the Adaboost had slightly higher test accuracy than the Random Forest model, their sensitivity was much lower, which predicts fewer patients with VTE, contrary to our aim. Even though Logistic Regression, on the other hand, had a higher sensitivity, the low specificity and AUPRC meant that too many patients without VTE were incorrectly predicted to have VTE. Therefore, we recommended the Random Forest model as the best classifier for this VTE prediction task.

**TABLE 2:** Evaluation measurements and 95% CI performed on the testing data.

Model	Accuracy	Sensitivity	Specificity	AUROC	AUPRC
Random forest	0.957 (0.934–0.975)	0.714 (0.619–0.762)	0.965 (0.941–0.985)	0.91 (0.893–0.926)	0.43 (0.363–0.500)
Adaboost	0.972 (0.954–0.974)	0.619 (0.381–0.714)	0.983 (0.968–0.986)	0.83 (0.701–0.926)	0.39 (0.230–0.476)
Xgboost	0.971 (0.965–0.976)	0.571 (0.429–0.667)	0.983 (0.977–0.989)	0.89 (0.861–0.937)	0.41 (0.323–0.470)
Logistic regression	0.954 (0.922–0.963)	0.761 (0.619–0.762)	0.961 (0.927–0.971)	0.90 (0.830–0.932)	0.37 (0.294–0.465)
KNN	0.901 (0.882–0.931)	0.190 (0.190–0.524)	0.924 (0.901–0.948)	0.73 (0.615–0.785)	0.16 (0.090–0.270)

*KNN, K-NearestNeighbor.*

**FIGURE 1:**
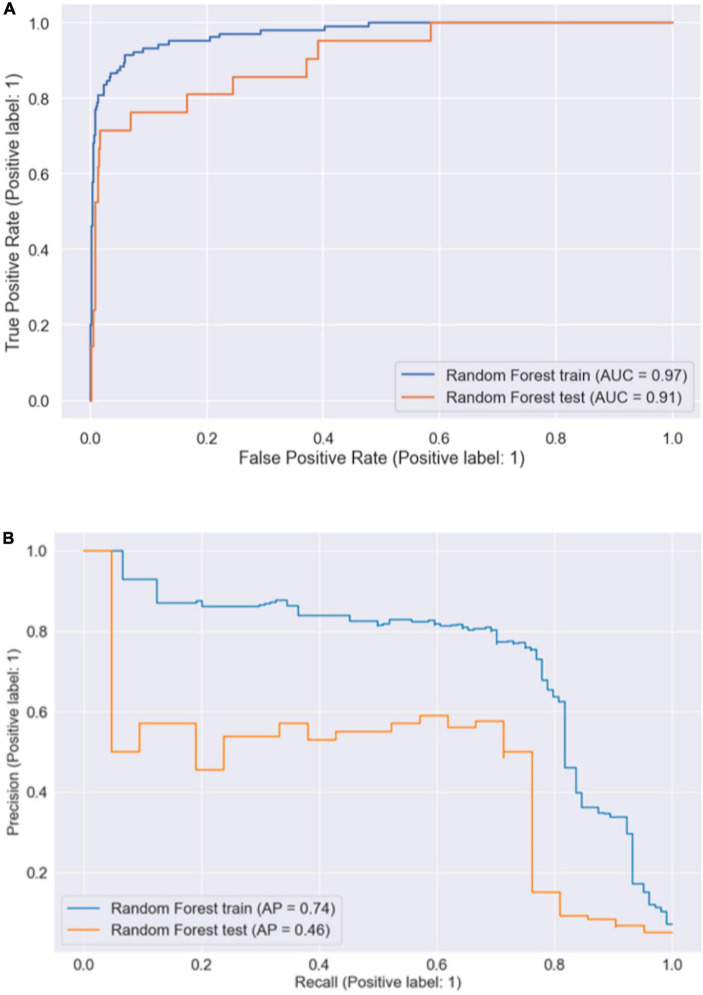
The receiver operating characteristic curve and the precision–recall curve on training and test dataset. **(A)** AUROC. **(B)** AUPRC.

**FIGURE 2:**
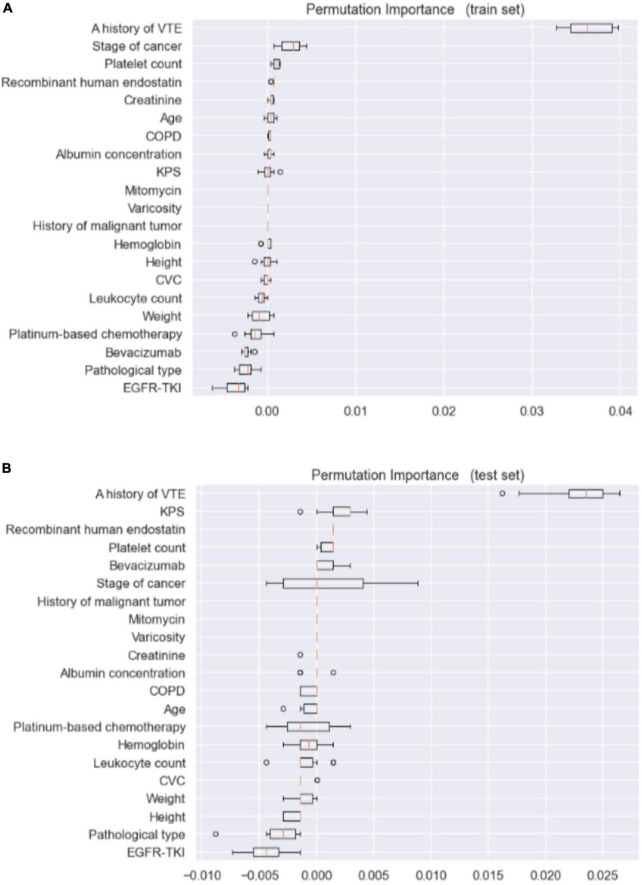
Permutation feature importance ranking for both training and test datasets. **(A)** Train set. **(B)** Test set.

**FIGURE 3:**
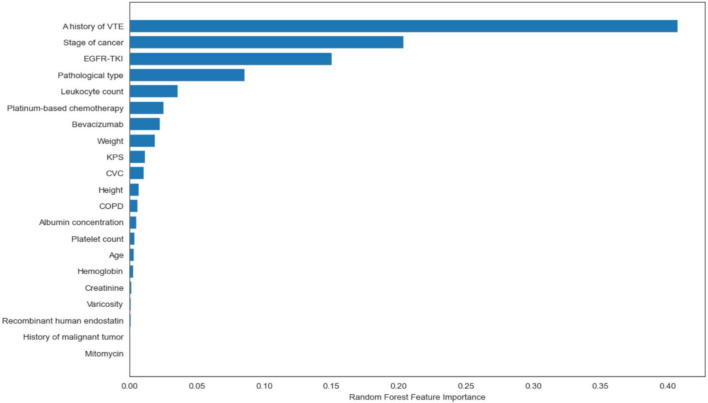
Impurity-based feature importance ranking.

Figure 1 shows the ROC curve and precision–recall curve for the Random Forest model, with an AUROC of 0.91 (95% *CI*: 0.893–0.926) and an AUPRC of 0.43 (95% *CI*: 0.363–0.500). Random forest model has a test accuracy score of 0.957 (95% *CI*: 0.934–0.975), sensitivity of 0.714 (95% *CI*: 0.614–0.762) and specificity of 0.965 (95% *CI*: 0.941–0.985). Besides the model performance, we ranked features according to their permutation feature importance during both the training stage and the test stage, as shown in Figure 2. The intersection of the top ten features consists of the following features: A history of VTE, Creatinine, KPS, Mitomycin, Platelet count, Recombinant human endostatin, and Stage of cancer. A feature of importance ranking was also performed using the random forest’s impurity-based score, as described in Figure 3. By taking another intersection with the top 10 features in this ranking, we regarded a history of VTE, KPS, and stage of cancer as the most contributing features.

**FIGURE 4:**
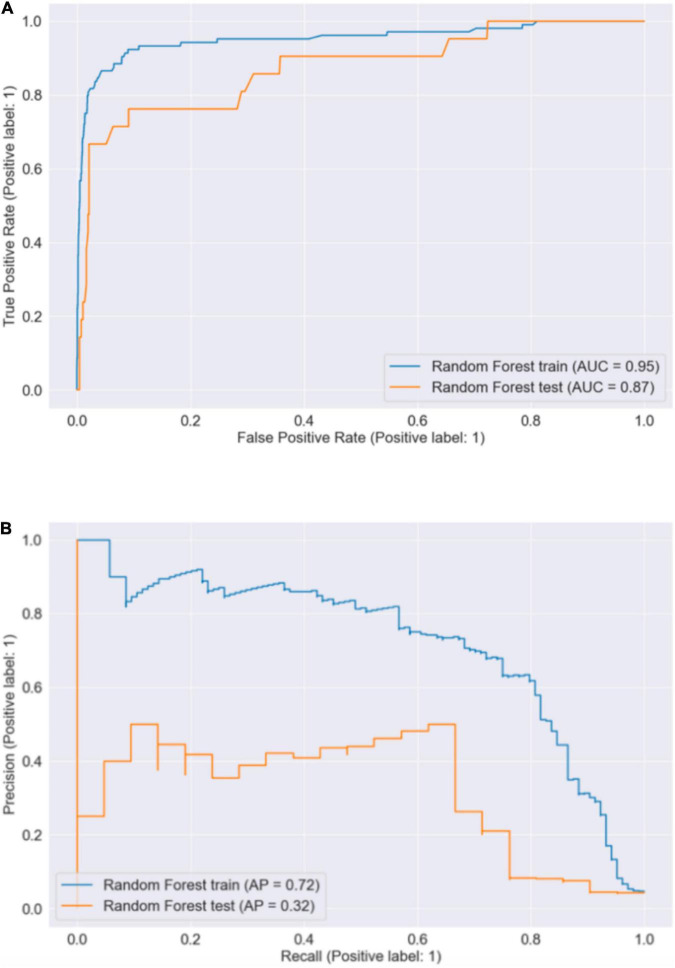
The receiver operating characteristic curve and the precision-recall curve on train and test dataset after feature selection. **(A)** AUROC. **(B)** AUPRC.

To generate a more easy-to-use model with less required input features, we selected the following five features: KPS, A history of VTE, Recombinant human endostatin, EGFR-TKI and Platelet count, and re-trained a random forest classifier. These five features were obtained by taking the union of the top three features from each of the three rankings (train permutation importance, test permutation importance, and impurity-based importance) mentioned above, with “Stage of Cancer” being removed because of its high missing rate. As shown in Figure 4, the AUROC and AUPRC achieved by this model were 0.87 and 0.30, respectively, with 95% *CI* of 0.802–0.917 and 0.265 and 0.358. Compared to Figure 1, there is no significant increase or decrease in these two performance assessment measures.

In addition, this model’s sensitivity score of 0.714 did not change (0.619–0.762). Yet, both the test accuracy score (0.929) and the specificity scores (0.936) were lowered, and their *Cis* were widened to the left direction, which were 0.896–0.968 and 0.900–0.977, respectively. Since there was no conspicuous decrease in the model’s performance, with the fact that it requires fewer features to predict, we conclude that it would be more applicable in real-life clinical settings.

## Discussion

A simplified and applicable ML model with five most relevant features, KPS, A history of VTE, Recombinant human endostatin, EGFR-TKI, and Platelet count, to predict VTE incidence in lung cancer patients was developed and validated in this study. In terms of the importance ranking of variables, our results are consistent with previous studies.

The published data have suggested that prior history of VTE history of VTE increases the risk of VTE recurrence by 2 to 7-fold after cancer diagnosis (21). Also, Sargam’s study showed the incidence of cancer-related VTE at 180 days was 10-fold higher in those with prior history of VTE compared to those without (36.9 vs. 3.66%; *OR*: 15.4, 95% *CI*: 15.22–15.6) in a cohort study of 4,159,400 patients.

The KPS is a simple and rapid method to assess patients’ performance in activities of daily living which has been mostly used in medical oncology (22). It is consensus that bedfast patients have higher risks of lower extremity VTE (23).

The EGFR-TKI has long been the first-line treatment for patients with EGFR-mutant NSCLC. Erlotinib, Icotinib, and Gefitinib have been tied to a significant increase in VTE. EGFR-TKI contributes to thrombosis, perhaps through endothelial cell and platelet activation (24). Yang A analyzed a total of 1,001 lung cancer retrospectively, and showed that the *HR* of VTE occurrence is 2.808 (95% *CI*: 1.439–5.479, *p* = 0.002) in patients with EGFR-TKI treatment relative to patients without the treatment (25).

Pedersen and Milman examined data on platelet counts obtained in a large population (1,178 patients) of patients with primary lung cancer. A high prevalence (32%) of elevated platelet counts was observed in patients with lung cancer (26).

Recombinant human endostatin is a kind of the antiangiogenic drugs independently developed in our country and has been suggested to treat NSCLC. It inhibits angiogenesis by blocking the pro-angiogenic activities of vascular endothelial growth factor (VEGF) and fibroblast growth factors (FGF)-basic, which is a risk factor for thrombosis (27).

Regarding ML models, in this specific study and dataset with a limited number of patients and variables, deep learning models were not used because the possibility of overfitting is high for such complicated models. In addition, there are many ways to evaluate a model’s performance, which depends on the dataset and its usage, which makes determining the better model difficult. In this study, we chose a random forest model for the following reasons.

The random forest classifier consists of an ensemble of decision trees and is based on the bagging method. This means that the model trains with a set of decision trees and takes the main voting to be the final output. The advantage of this method is that it is very stable and it can reduce the chance of overfitting, which is suitable for our relatively small dataset. This would, therefore, increase not only the test accuracy, but also the accuracy of the potential patients’ samples when the model is used in the future. Although it is widely accepted that random forest classifiers can produce good results without systematically selecting hyperparameters, we still performed hyperparameter tuning to further avoid overfitting and to increase the true positive rate.

This study, to our knowledge, is by far a novel study for a prediction model using ML method for VTE prediction in Chinese patients of lung cancer. One of its great strengths is in the large number of patients included in a real-world setting. The another strength of this study is that we selected five easily available variables to build the model, which has strong clinical applicability.

However, we acknowledge the following limitations. First, some patient information may not be completely collected, for example, some VTE risk factors were recorded in clinical notes (e.g., history of heart failure, surgery and fracture within 1 month before admission, and family history of VTE), which need specific natural language processing tool for information extraction, this part of data may be included in the modeling after data are well curated in future. Secondly, though some previous studies have shown that adenocarcinoma and VTE were more closely related than patients with squamous cell lung carcinoma, due to data incompleteness on the pathological types in this study, adenocarcinoma was classified into NSCLC. Thirdly, this study was a single-center, retrospective study using real-world data, which inevitably leads to bias. Another, in terms of the criteria of choosing risk factors, since acute comorbidities are hard to confirm through the timeline in this dataset, such as surgery or fracture and heart failure within 1 month, we chose chronic comorbidities for risk factors. Besides, our model still needs to be verified in multi-center populations prospectively in the future.

## Conclusion

In this retrospective, real-world, Chinese cohort study, we developed and validated a novel model using ML algorithms with minimal model input of 5 commonly used clinical variables, with an overall good performance AUROC of 0.87, AUPRC of 0.30, accuracy of 0.929, sensitivity of 0.714,and specificity of 0.936, allow identifying high-risk populations and preventing the development of VTE in patients with lung cancer.

## Data Availability Statement

The raw data supporting the conclusions of this article will be made available by the authors, without undue reservation.

## Author Contributions

HLei, HLiu, YW, and NH conceived and designed the study. CL, MZ, and XL collected and assembled data and were responsible for privacy management. ZW and WZ cleaned the data, analyzed and implemented the algorithm. BL, MG, GW, and YW interpreted the data. JM and HZ were responsible for literature review. HLei, MZ, and ZW drafted the manuscript. All authors revised the manuscript for important intellectual content.

## Conflict of Interest

MZ, ZW, CL, JM, HZ, MG, and NH was employed by company Digital Health China. The remaining authors declare that the research was conducted in the absence of any commercial or financial relationships that could be construed as a potential conflict of interest.

## Publisher’s Note

All claims expressed in this article are solely those of the authors and do not necessarily represent those of their affiliated organizations, or those of the publisher, the editors and the reviewers. Any product that may be evaluated in this article, or claim that may be made by its manufacturer, is not guaranteed or endorsed by the publisher.
