# Supplementary material for: Development and Validation of a Risk Prediction Model for Venous Thromboembolism in Lung Cancer Patients Using Machine Learning
Source: Front Cardiovasc Med. 2022 Mar 7;9:845210. doi: 10.3389/fcvm.2022.845210 (PMC8934875; doi:10.3389/fcvm.2022.845210)

Supplementary Figure S1: Data distribution of categorical data

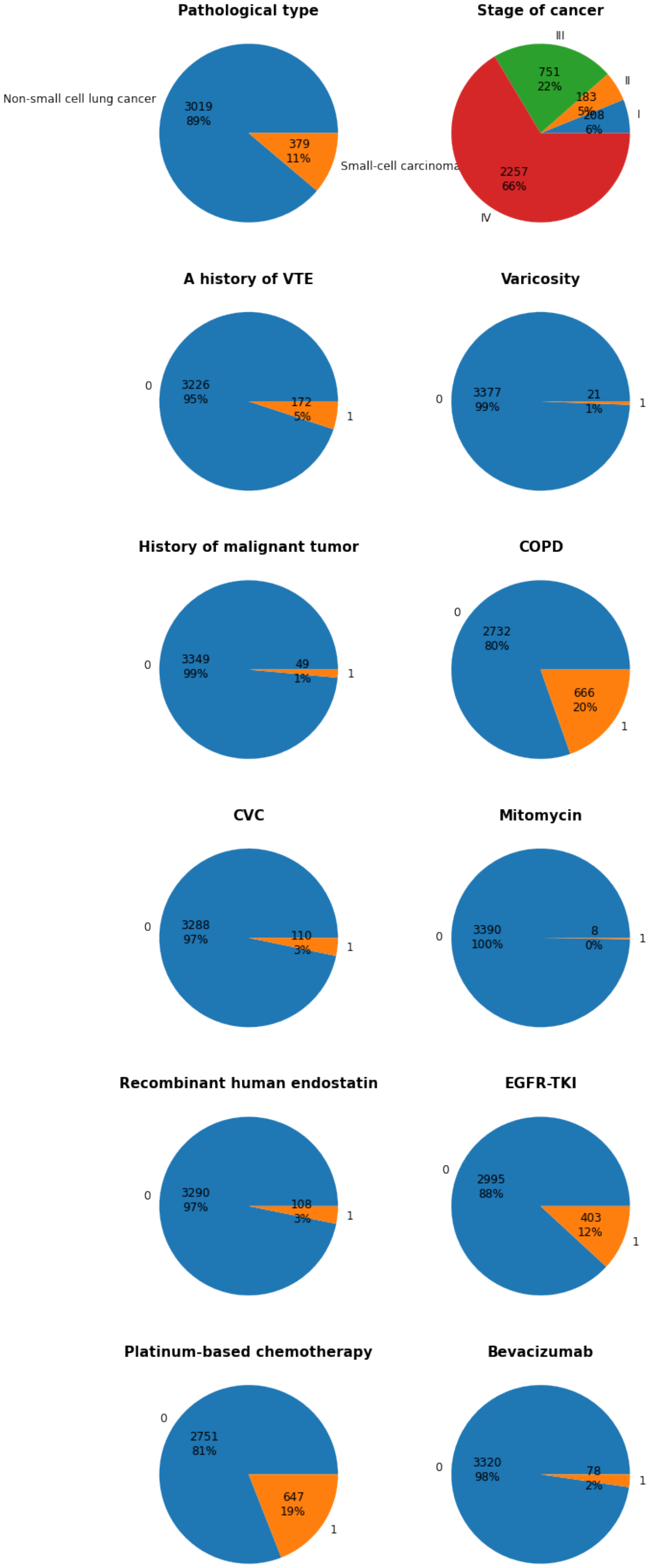

Supplementary Figure S2: Data distribution of continuous data

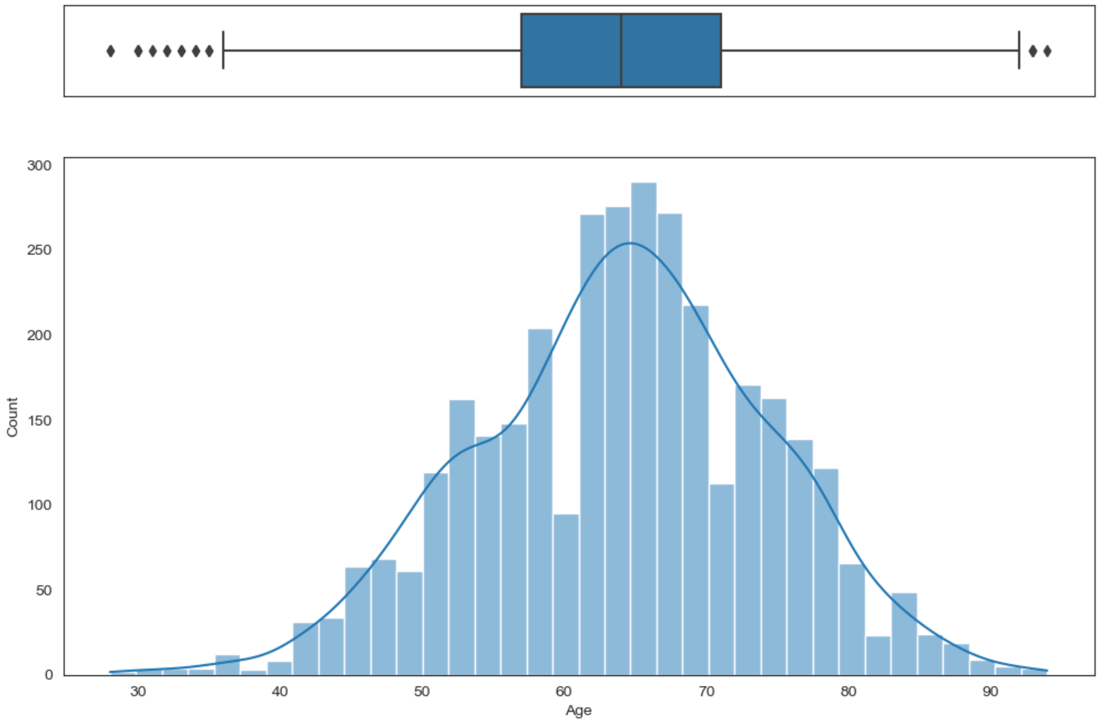

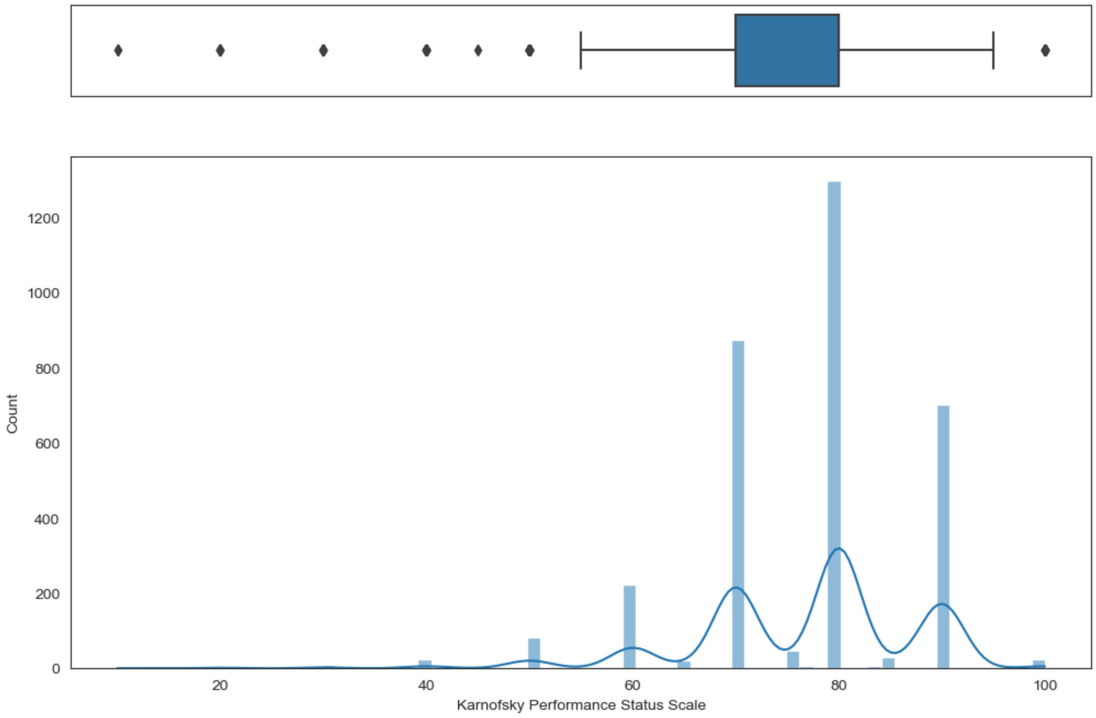

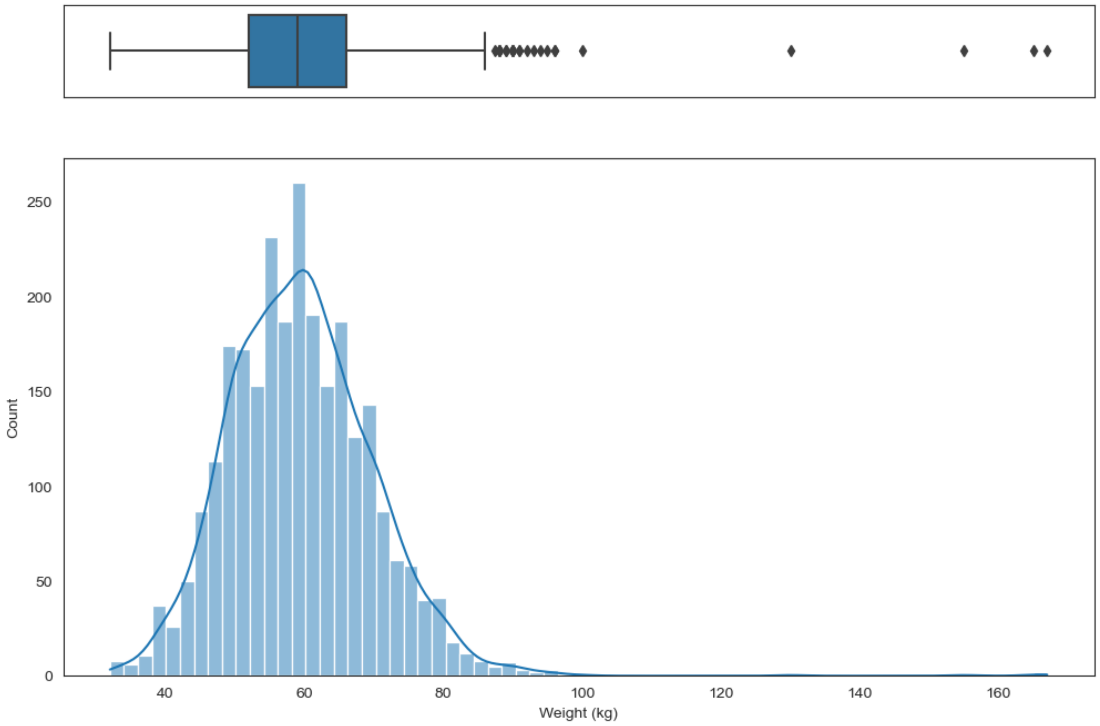
Count
0
50
100
150
200
250
40
60
80
100
120
140
160
Weight (kg)

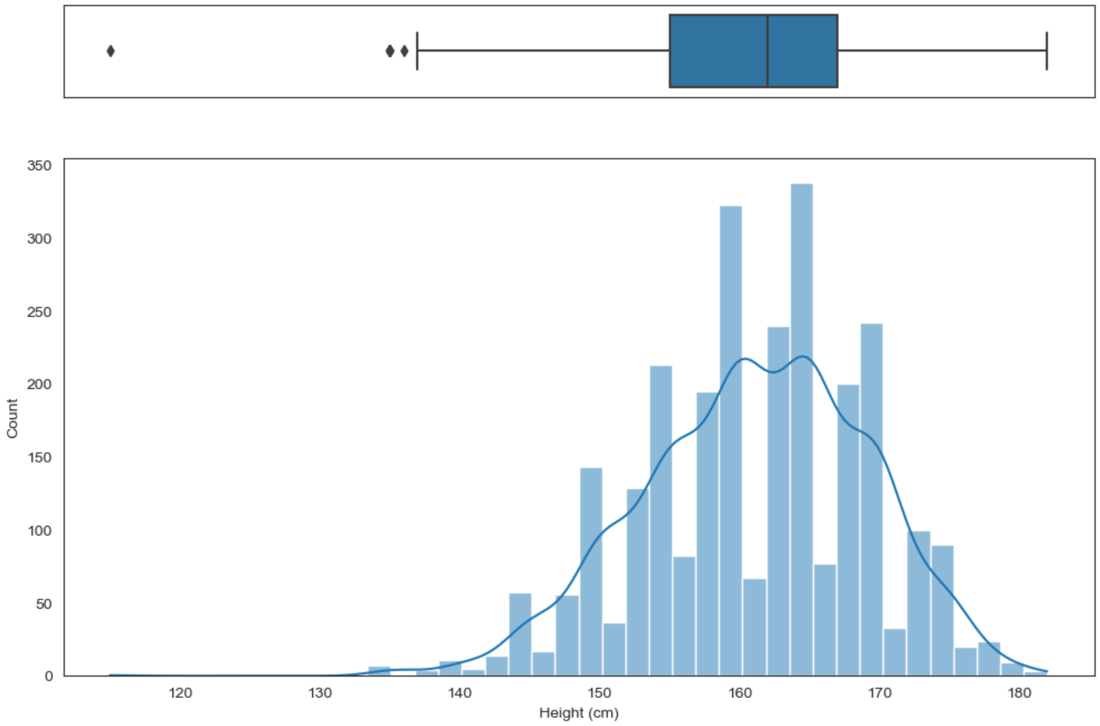
Count
0
50
100
150
200
250
300
350
120
130
140
150
160
170
180
Height (cm)

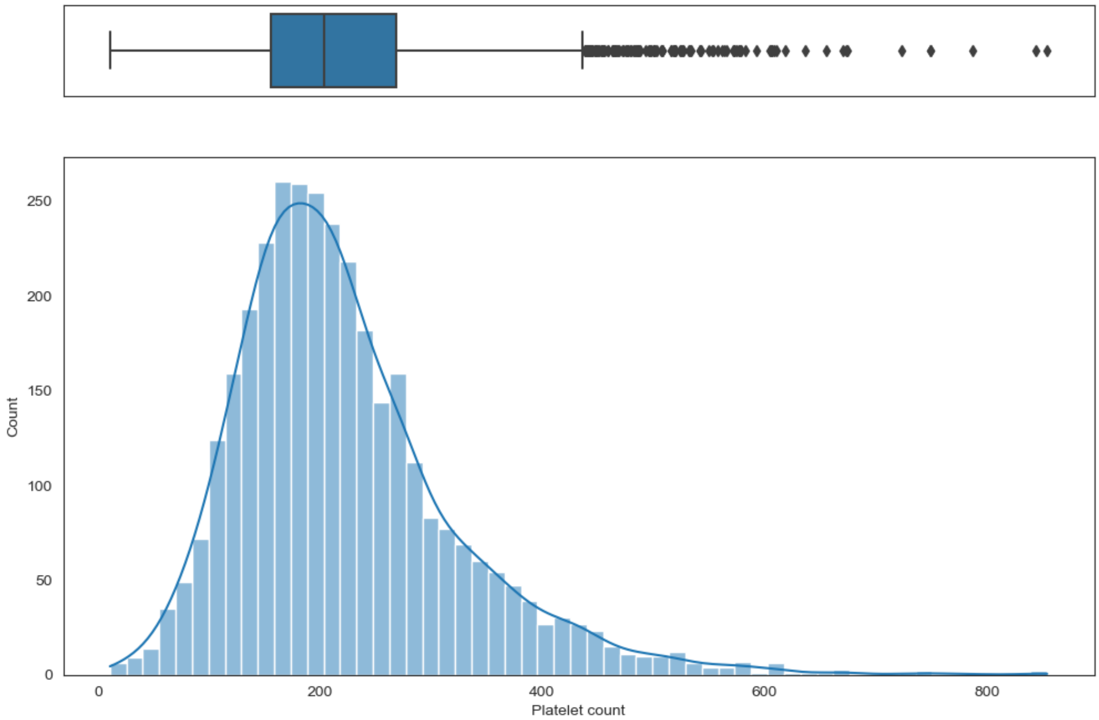

Count
250
200
150
100
50
0
0
200
400
600
800
Platelet count

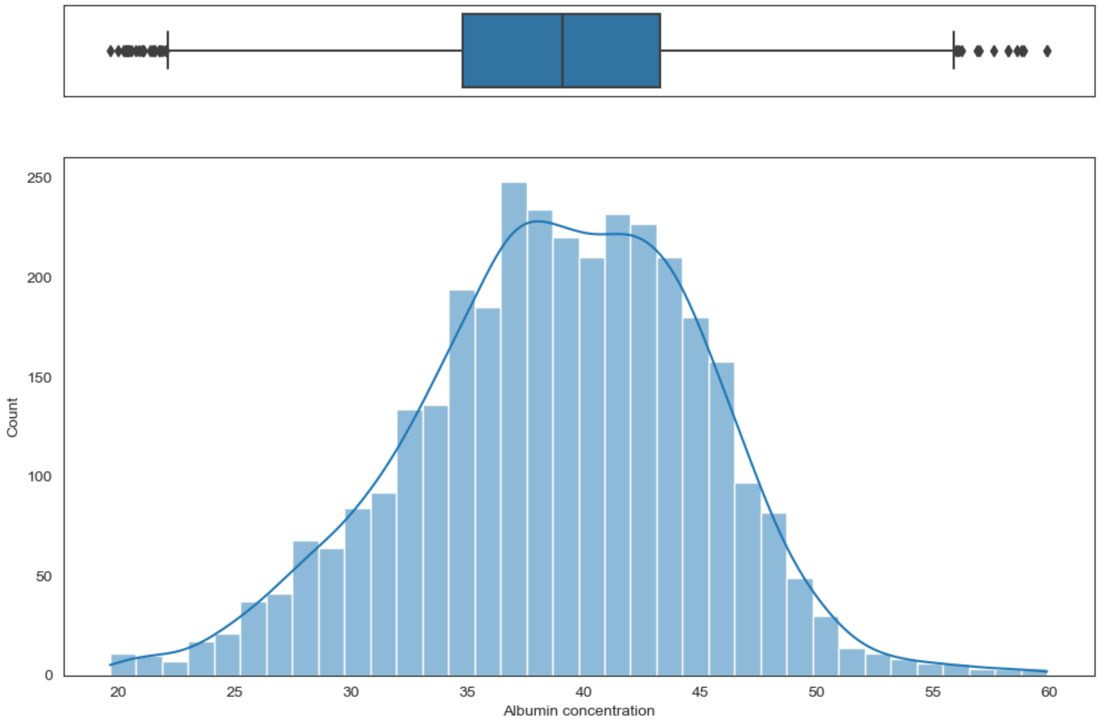

Count
250
200
150
100
50
0
20
25
30
35
40
45
50
55
60
Albumin concentration

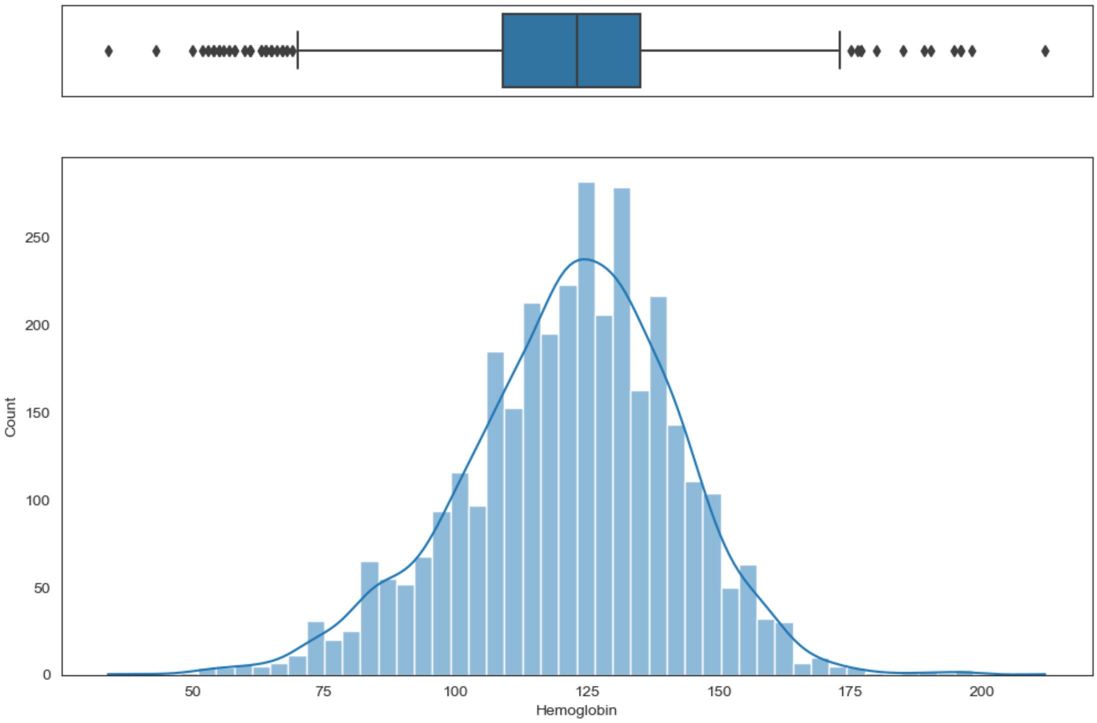
Count
0
50
100
150
200
250
50
75
100
125
150
175
200
Hemoglobin

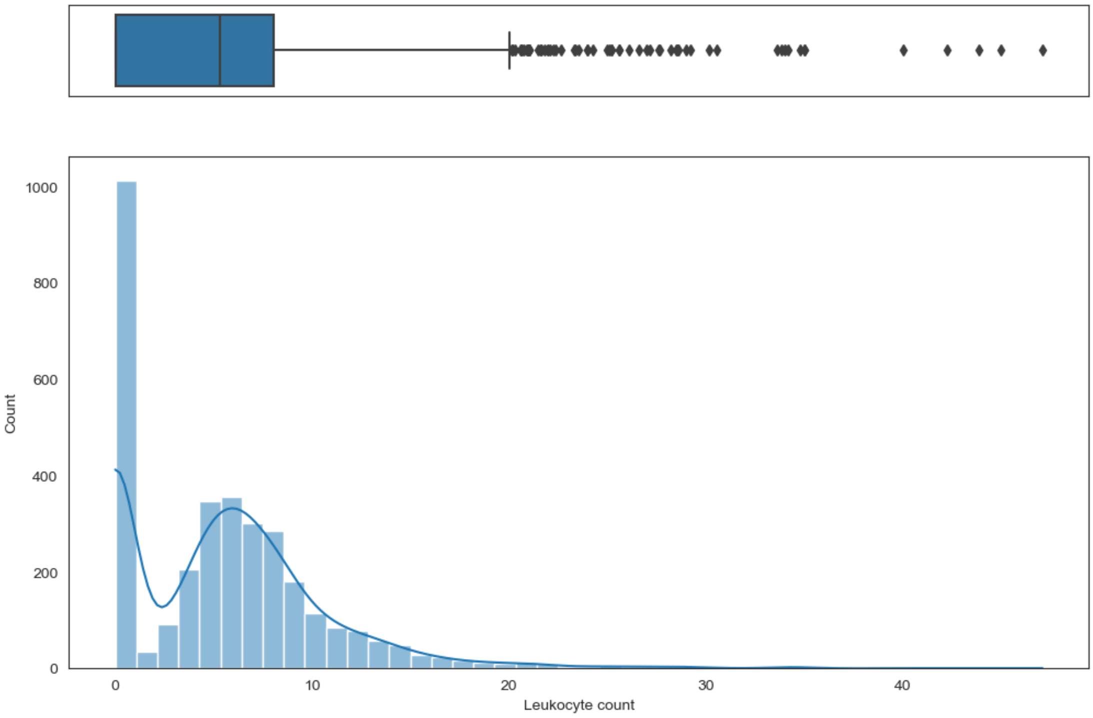
Count
0
200
400
600
800
1000
0
10
20
30
40
Leukocyte count

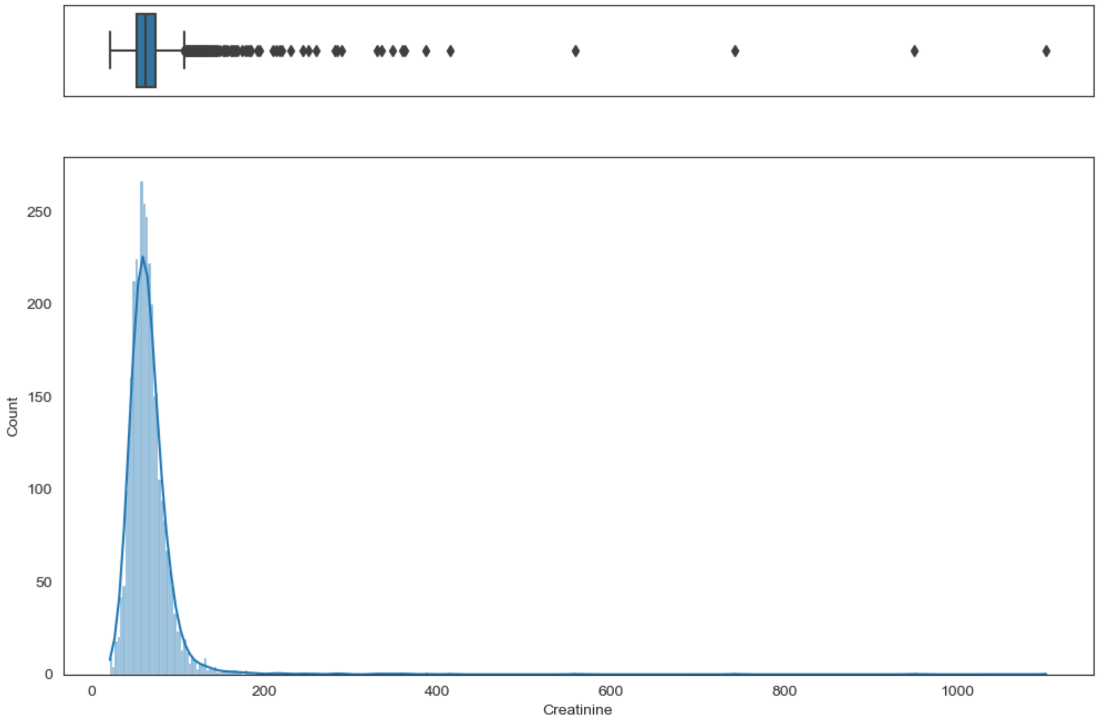
250
200
150
100
50
0
Count
0
200
400
600
800
1000
Creatinine

Supplementary Figure S3: Missing values

a) Location of all missing values with a dataset sorted by age
Missing values are represented with white bars. We can see that there is no particular pattern of missing values associate with age.

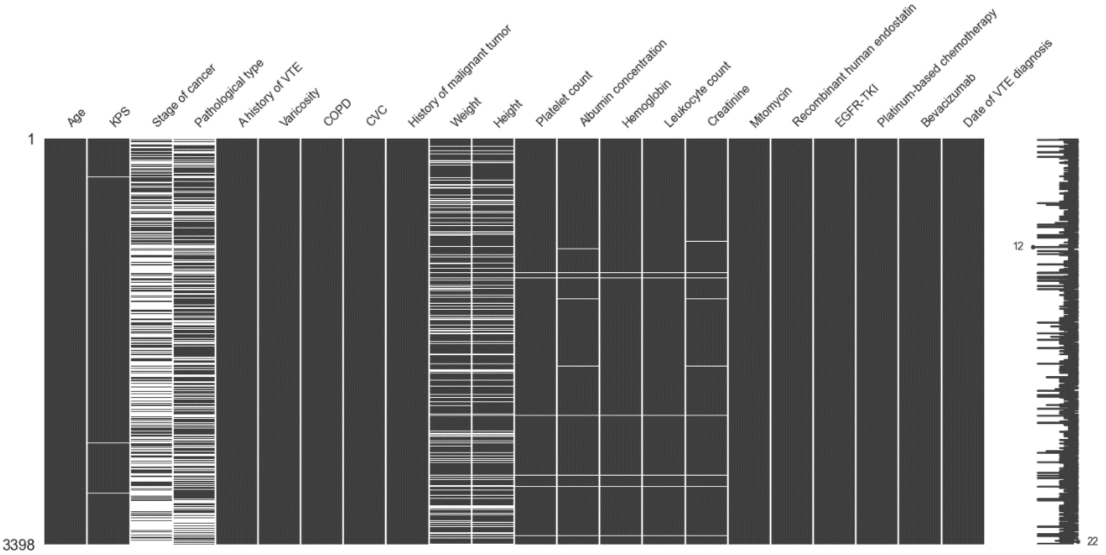

b) Percentage and absolute count of non-missing values for all features
Left y-axis shows the percentage and right y-axis shows the absolute count.

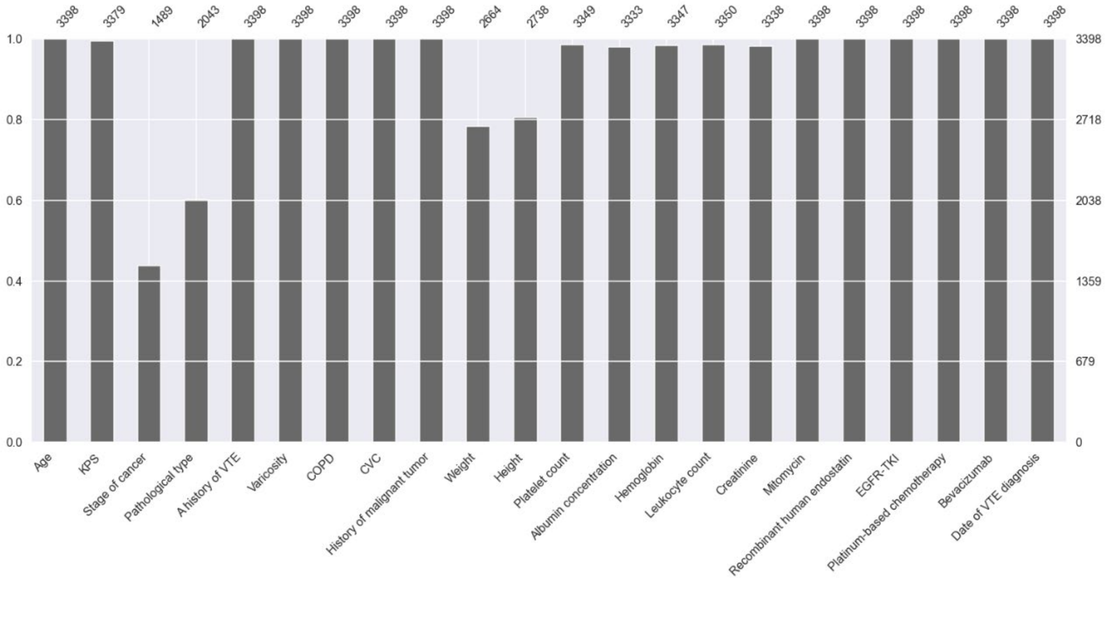

Supplement: Supplementary file 1 [file Data_Sheet_1.pdf]
